# Pennycress, carbon wise: labeling experiments reveal how pennycress seeds efficiently incorporate carbon into biomass

**DOI:** 10.1093/jxb/eraa136

**Published:** 2020-05-30

**Authors:** John C Sedbrook, Timothy P Durrett

**Affiliations:** 1 School of Biological Sciences, Illinois State University, Normal, IL, USA; 2 Department of Biochemistry and Molecular Biophysics, Kansas State University, Manhattan, KS, USA

**Keywords:** ^13^C-labeling, carbon conversion efficiency, isocitrate dehydrogenase, jet fuel, oilseed, pennycress, plant metabolism, Rubisco

## Abstract

This article comments on:

**Tsogtbaatar E, Cocuron J-C, Alonso AP. 2020**. Non-conventional pathways enable pennycress (*Thlaspi arvense* L.) embryos to achieve high biosynthetic efficiency. Journal of Experimental Botany **71**, 3037–3051.

**Pennycress (*Thlaspi arvense* L.) is being developed as an oilseed cash cover crop for producing biofuels and value-added products as part of a sustainable carbon-neutral future. An improved understanding of how pennycress embryos allocate photosynthate to biomass would help guide efforts to maximize and tailor seed oil production. To that end, Tsogtbaatar *et al.* (2020) fed pennycress embryos either ^13^C-labeled glucose or ^13^C-labeled glutamine, then quantified central carbon metabolism intermediates. Their data revealed that pennycress embryos are extremely efficient at converting substrates into biomass, due to key enzymatic activities that enable recapture of CO_2_, including the reversibility of isocitrate dehydrogenase and CO_2_ refixation by Rubisco.**

A preponderance of scientific evidence makes clear that humanity must transition to carbon neutrality by 2050, if not sooner, to avoid the worst effects of climate change on civilization and ecosystems (Masson-Delmotte *et al*., 2018). To do so, not only must the use of fossil fuels be curtailed and replaced, for example by using biofuels, but carbon capture and storage (CCS) will be necessary to return atmospheric CO_2_ to near current levels (Edenhofer *et al*., 2011, 2014).

An attractive route to increasing atmospheric carbon capture and biofuel production, without displacing food crops or disrupting ecosystems, is to grow cover crops in the off season on farmland otherwise sitting barren and prone to soil erosion and nutrient runoff (Phippen and Phippen, 2012; Dunn *et al.*, 2016). To that end, researchers are domesticating pennycress (*Thlaspi arvense* L.; field pennycress) (Box 1) into an oilseed cash cover crop grown during the autumn through spring months throughout temperate regions of the world (Sedbrook *et al.*, 2014; Chopra *et al.*, 2020). At a seed yield of 1700 kg ha^–1^ (projected to be economically profitable and what breeding programs are now attaining) and a harvest index of 0.3, pennycress plants would fix ~0.4 t of carbon per hectare annually (McDermitt and Loomis, 1981) while producing 600 liters of oil ha^–1^. If planted on half of the 32.4 Mha US Midwest Corn Belt (Fan *et al.*, 2013), pennycress could annually fix 40 Mt of carbon (the emissions of ~31 million automobiles) and produce 9.8 billion liters of oil and 17.5 Mkg of seed meal.

Box 1. **Engineering pennycress for higher seed oil content and improved composition.**

(A) Pennycress wild-type (WT) and *transparent testa 8* (*tt8*) plants growing in a field in central Illinois, USA. *TT8* encodes a transcription factor shown in Arabidopsis to negatively regulate fatty acid biosynthesis (Chen *et al.*, 2014). (B) Representative WT (left) and *tt8* mutant (right) seeds. Our unpublished data show that *tt8* seeds contain relatively less fiber and more oil and protein, signifying reallocation of photosynthate.

Erucic acid (22:1), a very long chain fatty acid, is derived from oleic acid (18:1) through the enzymatic activity of FATTY ACID ELONGASE 1 (FAE1) in the cytosol. Based on our published (McGinn *et al*., 2018) and unpublished data, pennycress *fae1* mutant seeds, while containing the desired abolishment of erucic acid in the oil, also contain significantly less total oil compared with the WT, signifying an undesirable disruption in photosynthate flux to oil. Analyses such as those detailed by Tsogtbaatar and co-workers will be useful in determining how metabolites flux differently in various genetic backgrounds and environmental conditions, and how improvements to seed oil content and composition can be optimized by mitigating introduced inefficiencies.

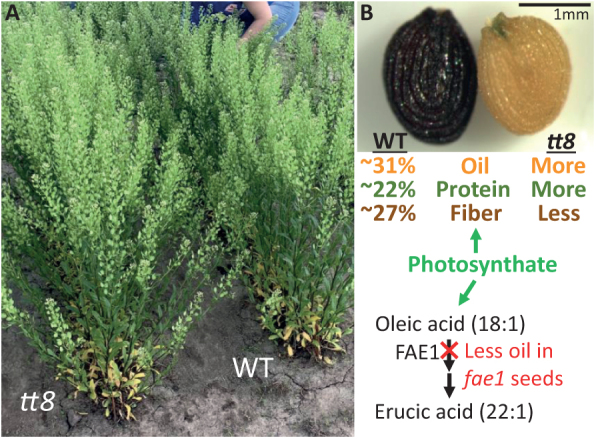

While native pennycress oil is suitable for conversion to biodiesel and biojet fuel (Moser, 2012; Fan *et al.*, 2013), the oil composition is being improved upon for various applications. For example, mutations in *FATTY ACID ELONGATION1* (*FAE1*) abolish seed oil erucic acid (22:1) content (Box 1), thereby improving the cold flow properties for biodiesel. Mutations that increase oleic acid content improve oil stability and shelf life in that this monounsaturated fatty acid is more oxidatively stable than the abundant polyunsaturated fatty acids linoleic (18:2) and linolenic (18:3) acids in native pennycress oil (Durrett *et al.*, 2008).

Wild pennycress seeds contain ~30–35% oil on a dry weight basis. Given the value of higher oil content and the fact that the indigestible seed coat fiber (composed of structural polysaccharides, condensed tannins, and lignin) must be reduced to improve the nutritional value of the seed, breeding and metabolic engineering efforts are underway to redirect carbon in developing seeds (Chopra *et al.*, 2018). To help guide these efforts, Tsogtbaatar *et al.* (2020) have provided the first quantitative assessment of carbon uptake and utilization by developing pennycress embryos. By measuring the composition of the liquid endosperm that sustains embryo development, they established *in vivo* embryo culture conditions that enabled similar physiological development and biomass accumulation rates to embryos *in planta*. With this culture system, Tsogtbaatar and co-workers measured the uptake and incorporation of carbon by pennycress embryos and demonstrated a carbon conversion efficiency (CCE) of 93.4%, which is considerably higher than that observed for other oilseeds. For example, other Brassicaceae such as *Brassica napus* (oilseed rape) and *Camelina sativa* (camelina) possess a CCE of 86% and just 32%, respectively (Goffman *et al.*, 2005; Carey *et al.*, 2020).

## Distinct pathways enable efficient CO_2_ capture

How is pennycress able to achieve such a high CCE compared with related species? Follow-up experiments by Tsogtbaatar *et al.* (2020) suggest at least two mechanisms, in two different organelles, by which pennycress embryos are able to efficiently re-incorporate the CO_2_ generated when pyruvate is decarboxylated to generate acetyl-CoA in the mitochondria and plastids (Box 2).

Box 2. **Two different pathways enable efficient CO_2_ incorporation into seed biomass.**

A number of metabolic reactions necessary for the synthesis of oil, such as the decarboxylation of pyruvate to form acetyl-CoA, generate CO_2_ and thus waste carbon derived from photosynthate. Developing pennycress seeds are able to recapture the CO_2_ that is produced during metabolism. Both the mitochondria and chloroplasts possess reactions that enable this efficient use of carbon. In chloroplasts, Rubisco fixes CO_2_ to form 3-phosphoglycerate (PGA) which, in the absence of the Calvin cycle, can be converted to acetyl-CoA and used to synthesize fatty acids. In mitochondria, the reverse action of isocitrate dehydrogenase (IDH) enables the carboxylation of α-ketoglutarate to form citrate. Once exported to the cytosol, the citrate is converted to acetyl-CoA which can be used by the fatty acid elongase complex to synthesize erucic acid (22:1) from oleic acid (18:1).

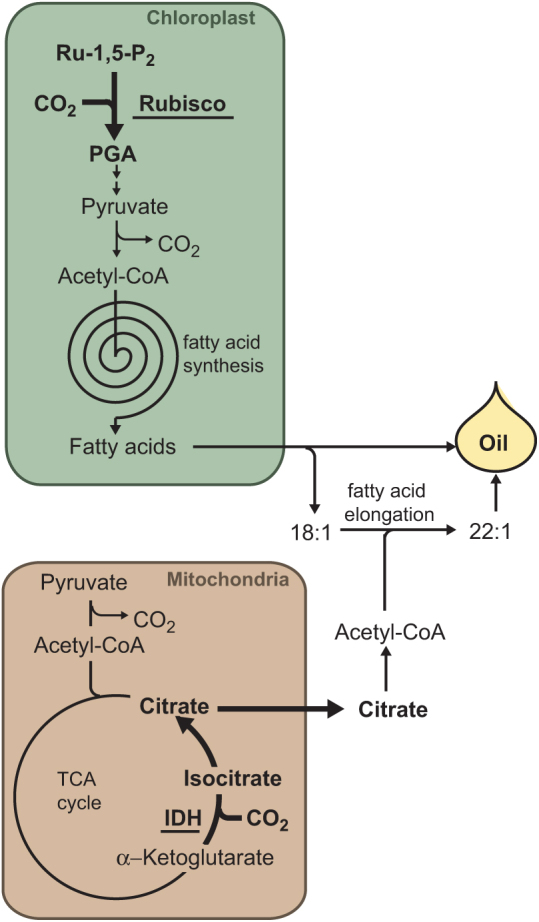

One mechanism, first demonstrated through pioneering stable isotope labeling studies in *Brassica napus* (Schwender *et al.*, 2004), involves the refixation of CO_2_ by Rubisco to form 3-phosphoglycerate (PGA) (Box 2). In the absence of the remainder of the Calvin cycle, this PGA is ultimately converted to acetyl-CoA and used to synthesize fatty acids. In *B. napus*, this so-called ‘Rubisco shunt’ is estimated to generate 37–51% of the PGA in the embryo, thus reducing carbon loss and contributing to the high CCE of this species. Other seeds with relatively high CCE, such as soybean, also appear to utilize the Rubisco shunt (Allen *et al.*, 2009). In contrast, recent work has shown that developing camelina embryos lack this particular capability and thus possess a much lower CCE of 32% (Carey *et al.*, 2020). With a similar stable isotope labeling strategy, Tsogtbaatar *et al.* (2020) were able to show that like *B. napus*, pennycress embryos also use Rubisco to recapture CO_2_, forming ~25% of PGA in this manner.

In addition to the Rubsico shunt, pennycress embryos also appear able to recapture CO_2_ through the reverse action of isocitrate dehydrogenase in the tricarboxylic acid (TCA) cycle (Box 2). Typically, this enzyme functions to decarboxylate isocitrate (formed from citrate) to α-ketoglutarate. However, Tsogtbaatar and co-workers demonstrate that when they label pennycress embryos with [U-^13^C_5_]glutamine, a significant proportion of the citrate and isocitrate formed is labeled on five carbon atoms. As the carbon backbone of the labeled glutamine enters the citric acid cycle as α-ketoglutarate, the forward action of the citric cycle would result in molecules labeled on four carbons due to the decarboxylation of α-ketoglutarate. Consequently, the only way to facilitate the labeling of five carbon atoms of citrate is to directly convert the labeled α-ketoglutarate to isocitrate with the concomitant incorporation of CO_2_, through the reverse action of isocitrate dehydrogenase (Box 2). The reverse action of this enzyme in plants is not without precedent, as it has also been demonstrated to occur in developing *B. napus* and soybean seeds (Schwender *et al.*, 2006; Allen *et al.*, 2009), where it presumably contributes to the higher CCE of these species.

## Provisions of substrates for fatty acid elongation

The labeling experiments of Tsogtbaatar *et al.* (2020) also reveal how different pathways might function to provide carbon and reductant for the synthesis of erucic acid. This very long chain monounsaturated fatty acid is synthesized in the cytosol by the fatty acid elongation complex which uses acetyl-CoA and NADPH to elongate oleoyl-CoA. Here, the reverse activity of isocitrate dehydrogenase is significant as it allows the increased production of citrate which can be exported from the mitochondria. In the cytosol, acetyl-CoA can be derived from this citrate through the action of citrate lyase, suggesting how additional 2C units can be provided for the synthesis of the large quantities of erucic acid produced in pennycress (Box 2).

Elongation of fatty acids also requires reductant. Here, labeling experiments using different [^13^C]glucose substrates demonstrated that the oxidative pentose phosphate pathway (OPPP) was active in developing pennycress seeds. The oxidative portion of this pathway is important for the generation of NADPH and can occur in both the cytosol and plastids. Careful analysis of hexose phosphate labeling patterns by Tsogtbaatar and co-workers suggested that in developing pennycress embryos the OPPP produces more NADPH in the cytosol, which they suggested is used to generate the abundant erucic acid found in wild-type pennycress oil.

## Implications for crop improvement

The insights generated by this work provide directions to improve the yield and composition of pennycress. One obvious implication is that given an already high CCE, increasing oil content will probably need to occur at the expense of either the protein, carbohydrate, and/or phenolic polymer components of the seed. As protein meal represents a valuable co-product, the extra carbon for increased oil content should preferably be derived from carbohydrate fractions such as cellulose or starch and phenolic polymer structural components including condensed tannins and lignin, which together comprise 36.3% of the carbon in the seed. Evidence for the viability of this approach already comes from pennycress *tt8* mutants, defective in a transcription factor shown in Arabidopsis to negatively regulate fatty acid synthesis (Chen *et al.*, 2014). Our unpublished results show that pennycress *tt8* mutants possess reduced seed coat fiber content and a relative increase in seed oil content without affecting plant growth and seed yield (Box 1).

Pennycress crop improvement efforts have included the elimination of erucic acid from the seed oil through the targeted mutagenesis of the *FAE1* gene using genome editing and ethyl methanesulfonate (EMS) mutagenesis approaches (McGinn *et al.*, 2019; Chopra *et al.*, 2020). The work of Tsogtbaatar *et al.* (2020) suggests that pennycress embryos possess pathways that devote carbon and reductant for the synthesis of erucic acid. It will therefore be interesting to determine at the gene and metabolite level how pennycress embryos respond to the disruption of erucic acid synthesis in the *fae1* mutant. What happens to the now unneeded acetyl-CoA and NADPH generated in the cytosol ostensibly for elongation? Our unpublished results indicate that *fae1* mutant seeds possess lower seed oil content compared with the wild type (Box 1), suggesting the introduction of metabolic inefficiencies into fatty acid synthesis. These could result from poor utilization of substrates, as has been suggested to occur in camelina where high flux through the OPPP lowers the CCE (Carey *et al.*, 2020). Alternatively, the aberrant accumulation of biosynthetic precursors could negatively regulate fatty acid production, as has been shown to occur during the synthesis of unusual fatty acids in transgenic plants (Bates *et al.*, 2014). Future work that builds on the approaches and results developed by Tsogtbaatar and co-workers will therefore provide new insights into how oil seeds regulate the flow of carbon into oil and other seed components.
